# The use and effects of synthetic cannabinoid receptor agonists by New South Wales cannabis treatment clients

**DOI:** 10.1186/s42238-021-00091-z

**Published:** 2021-07-26

**Authors:** Melissa A. Jackson, Amanda L. Brown, Jennifer Johnston, Richard Clancy, Iain McGregor, Raimondo Bruno, Nick Lintzeris, Mark Montebello, Jennifer Luksza, Jenny Bowman, Nghi Phung, Dave Allsop, Adrian J. Dunlop

**Affiliations:** 1 Drug and Alcohol Clinical Services, Hunter New England Local Health District, Level 3, 670 Hunter Street, Newcastle, NSW 2290 Australia; 2 University Centre for Rural Health, University of Sydney, Lismore, NSW Australia; 3 Centre for Brain and Mental Health Research, Hunter New England Local Health District, Newcastle, NSW Australia; 4 The Lambert Initiative for Cannabinoid Therapeutics, Brain and Mind Centre, University of Sydney, Sydney, NSW Australia; 5 School of Health, University of Tasmania, Hobart, TAS Australia; 6 Drug and Alcohol Services, South East Sydney Local Health District, Sydney, NSW Australia; 7 Drug and Alcohol Services, Northern Sydney Local Health District, Sydney, NSW Australia; 8 Drug Health, Western Sydney Local Health District, Sydney, NSW Australia; 9 Faculty of Science and Information Technology, University of Newcastle, Newcastle, NSW Australia; 10 Drug Health, Western Sydney Local Health District, Parramatta, NSW Australia

**Keywords:** Synthetic cannabinoid receptor agonist, Synthetic cannabinoids, Cannabis, Marijuana, Substance use disorder, Treatment

## Abstract

**Introduction:**

Despite decreasing consumption by general populations, use of synthetic cannabinoid receptor agonists (SCRAs) persists in some marginalised groups, including those who use other substances. This article explores SCRA consumption in an Australian cannabis treatment sample, comparing those who report ever using SCRAs with those who have never used SCRAs.

**Methods:**

A questionnaire orally administered in person to a convenience sample of 154 cannabis treatment service clients from New South Wales, Australia (71% male, median age 35) collected information regarding cannabis and SCRA use including motivations, effects and health-related consequences of use, demographics, other substance use and overall health. Demographic profiles and between-group differences were explored. McNemar tests compared effects of SCRA and cannabis. Logistic regression analysis determined predictors of SCRA use.

**Results:**

Half (53%) reported lifetime SCRA use; 20% reported previous-month use. The SCRA + cannabis group displayed greater polysubstance use and psychological distress. Reduced dependence on cannabis but higher levels of other substance use may predict SCRA use. Although curiosity motivated initial SCRA consumption, perceived psychoactive strength drove continued use. SCRAs appear to induce more negative side-effects than cannabis. Of the SCRA + cannabis group, 27% sought medical assistance for SCRA use. Most (90%) preferred cannabis to SCRAs, citing superior safety, effects and consistency of cannabis.

**Conclusions:**

Among clients seeking treatment for cannabis use, SCRA use was relatively common, although not a preferred substance. Hazardous substance use and poor mental health characterised SCRA consumers, highlighting the need for continued monitoring by researchers and treatment providers of SCRA consumption in populations who use substances.

**Supplementary Information:**

The online version contains supplementary material available at 10.1186/s42238-021-00091-z.

## Background

The past decade has seen the emergence of a novel class of drugs known collectively as New Psychoactive Substances (NPS). These have proliferated across global drug markets, posing considerable challenges to public health, law enforcement and drug policy (European Monitoring Centre for Drugs and Drug Addiction and Europol 2019). Synthetic cannabinoid receptor agonists (SCRAs) are currently the largest substance sub-category within the class, encompassing at least 180 distinct molecular entities (Potts et al. 2020). These laboratory-made chemical compounds were first introduced into the Western European recreational drug markets in 2004 after being found to mimic some effects of cannabis intoxication. Manufactured in powder form, SCRAs are typically mixed with organic plant material to create herbal smoking products that resemble cannabis itself. However, an increase in seizures of powder, tablet and liquid forms has been observed in recent years (European Monitoring Centre for Drugs and Drug Addiction 2017). Although they share some psychoactive similarities, SCRAs are structurally distinct from plant-based delta 9 tetrahydrocannabinol (Δ9-THC), a partial agonist of human CB1 and CB2 receptors. SCRA’s are generally full CB1 agonists (Seely et al. 2012), typically creating outcomes more rapid and intense than those experienced with cannabis (Fantegrossi et al. 2014). Cannabis also contains cannabidiol, a phytocannabinoid thought to be responsible for counteracting some of the harmful psychoactive effects of Δ9-THC (Swift et al. 2013). It is believed that similar protective agents lacking in SCRA’s potentially compound their harmful outcomes, particularly increasing the risk of psychosis in users (Spaderna et al. 2013; van Amsterdam et al. 2015).

SCRAs emerged as a drug of concern in the USA, Australia and New Zealand around 2010–2011 (Barratt et al. 2013). In Australia, attempts by state and federal regulators between 2012 and 2015 to schedule individual SCRA compounds did little to halt the chemical diversity and increasing number of new variants entering the market (Cairns et al. 2017). Federal drug analogue legislation was introduced in 2015 prohibiting all substances with chemical structures or psychoactive effects similar to those of previously controlled psychoactive drugs (excluding alcohol, tobacco and food products). Several Australian states (New South Wales, Queensland, South Australia and Victoria) adopted blanket bans to prohibit SCRA use, possession, production and supply, while other states and territories continue to schedule individual NPS as they emerge (Grigg et al. 2020).

Similar drug laws have been introduced internationally to regulate SCRAs and NPS more broadly. In the USA, Federal laws have placed SCRAs in Schedule I of the Controlled Substances Act, with individual states banning some or all SCRA compounds (Cairns et al. 2017). In Canada, all substances that mimic cannabis are listed as a Schedule II drug under the Controlled Drugs and Substances Act S.C. 1996, c. 19. (Canada). Such laws have been effective in reducing their availability and use (European Monitoring Centre for Drugs and Drug Addiction 2018), while increases in harmful SCRA use in regions where individual scheduling continues (Grigg et al. 2020). The European Monitoring Centre for Drugs and Drug Addiction Early Warning System identified 215 novel NPS between 2015 and 2017, noting the first decrease in new SCRA identifications in 2017 (European Monitoring Centre for Drugs and Drug Addiction 2018). Despite this, SCRAs remain the most frequently seized NPS worldwide and their use in parallel with other substances, particularly by high-risk populations, remains evident (Blackman and Bradley 2017; Joseph et al. 2019).

### Who uses SCRAs?

Early SCRA demographic profiling suggested consumers were young, well-educated males with high rates of employment, who also used cannabis, alcohol and tobacco (Barratt et al. 2013; Winstock and Barratt 2013b; Vandrey et al. 2012). Changing legislation and increased awareness of SCRA harms has seen large declines in consumption. SCRA use is now more commonly reported in people involved in the criminal justice system who may be seeking to avoid drug detection (Smith et al. 2017) and in other groups exposed to risk including homeless people, those with a history of mental illness and other psychoactive substance consumers (Clancy et al. 2018; Joseph et al. 2019; Ralphs et al. 2017; Sutherland et al. 2016; Peacock et al. 2019). A 2016–2017 US sample from a community-based support organisation for people who use drugs described recent SCRA consumers as older males, commonly diagnosed with psychiatric illness, who report concomitant use of SCRA with other substances, particularly opiates (Joseph et al. 2019). In Australia, SCRA use is more commonly seen in people who use cannabis and/or methamphetamines, or who regularly use multiple substances, or who experience social disadvantage and report difficulties ceasing substance use (Sutherland et al. 2018; Manseau et al. 2017).

### Motivations to use SCRAs

Studies exploring motivations to use SCRAs have cited explanations including the desire for novel euphoric experiences and an alternative to cannabis. Other drivers draw on the novel characteristics of SCRAs including their accessibility, intense recreational effects, value for money and ability to evade detection in drug screening (Barratt et al. 2013; Vandrey et al. 2012; Bonar et al. 2014; Macgregor and Payne 2013). Early reports of motives for initial SCRA consumption suggested curiosity, availability and legal access (Barratt et al. 2013; Vandrey et al. 2012). Motivations vary according to the population under study: individuals enrolled in US-based residential substance use treatment programmes who used SCRAs, cited peer influence and the desire to avoid positive drug screenings (Smith and Staton 2019). Reasons behind continued SCRA use, or about the internal drivers of SCRA use, are not well understood.

### Health-related consequences of SCRA use

#### Physical and psychological effects

Awareness of the acute and long-term physical and psychological harms of SCRAs has developed in recent years. Their ability to produce euphoric effects similar to those of cannabis, but with a wider range of negative consequences, has been described (Fattore and Fratta 2011; Spaderna et al. 2013; van Amsterdam et al. 2015). Tachycardia, anxiety, agitation, paranoia and nausea are the most common adverse effects of SCRA intoxication (Tait et al. 2016). Those not often seen with cannabis include seizures, agitation, hypertension and hypokalaemia (Fattore 2016). More severe complications include seizures, acute kidney injury, cardiac arrest, mood disturbances, acute psychosis and death (Darke et al. 2020). Direct comparisons of positive and negative effect domains have revealed more positive effects and ability to function during cannabis intoxication, while SCRA intoxication provides more negative effects and hangover symptoms (Winstock and Barratt 2013).

### Treatment utilisation

A 2012 global anonymous, online assessment of drug use found past year SCRA consumers (*n* = 2176) to be 30 times more likely to require emergency medical treatment following use compared to individuals following cannabis use, demonstrating the elevated risk of short-term harm carried by acute SCRA use (Winstock et al. 2015). Survey data from 2011 indicated that 2.4% of past year SCRA consumers (*n* = 980) sought emergency medical treatment, presenting primarily for panic, anxiety, paranoia and breathing difficulties (Winstock and Barratt 2013a). Information from Internet-based substance use samples, however, may not be broadly representative of people who use cannabis.

### Aims of the current study

Despite an overall decrease in availability and use of SCRAs, consumption in marginalised groups, including individuals with a history of substance dependence or criminal justice system involvement and those who experience social disadvantage, is still a concern. The current study will add to the existing body of SCRA literature by providing the first exploration of SCRA consumption in an Australian cannabis treatment sample. Its aims are to:

1. Compare groups of people in treatment for cannabis use—those who report *ever* using SCRAs with those who have *never* used SCRAs—in terms of demographics, substance use and health
2. Examine the factors influencing decisions to use or not to use SCRAs
3. Explore the experiences and outcomes of SCRA use with those who report ever using them

## Methods

### Procedure

The study was coordinated by a drug and alcohol clinical research unit located within Hunter New England Local Health District and received ethical approval from South East Sydney Local Health District Human Research Ethics Committee (Ref no: 15/030 HREC/15/POWH/56) and the University of Newcastle (Ref: H-2015–0120).

Potential participants were over 18 years of age and seeking assistance to manage their cannabis use from specialised cannabis treatment clinics in five local health districts across metropolitan, regional and rural NSW. They were asked by treating clinicians about their interest in participating in a study describing their experiences with cannabis and SCRAs if they had used them, or their reasons for not trying SCRAs if they had not used them. If they agreed, they were referred to a research staff member at each site and a face-to-face interview was arranged. Informed consent was obtained in writing from participants prior to interview commencement. No details were recorded about those who declined a referral for the research interview. All interviews were completed between July 2015 and April 2016.

Participants were grouped according to self-reported SCRA consumption: those who had never used SCRAs (cannabis-only group) and those who had used both cannabis and SCRAs (SCRA + cannabis group). Interview length varied; the SCRA + cannabis group took approximately 1 h and the cannabis-only group, approximately 30 min**.** All participants were reimbursed with retail vouchers for the time associated with study participation: $40 for the SCRA + cannabis group and $20 for the cannabis only group.

### Measures

Self-reported responses were collected from both groups to the following items (where indicated, the SCRA + cannabis group completed equivalent items relating to SCRA use). See Supplementary Materials for more detailed instrument descriptions and evidence of validity.

### Demographic and substance use information

Age, gender, aboriginal status, education, relationship status, living arrangements, income source, substance use history (lifetime, 3- and 1-month use, and days per month), detailed cannabis and SCRA use were recorded. Cannabis dependence was assessed using ICD-10 criteria with dependence considered if at least three of six criteria had been experienced concurrently in the previous 12-month period.  (World Health Organization 1992). 

### Effects of cannabis/SCRA use

Thirty-five physiological, psychological and behavioural consequences of cannabis and SCRA use were rated by the extent to which respondents had experienced each of them while intoxicated (1 = never, 2 = sometimes, 3 = most of the time, 4 = every time). Designed for the current study, the survey incorporated a range of effects (e.g. felt stimulated/energetic; increased ability to function; felt nervous or anxious).

### Drug Use Motive Questionnaire (DUMQ): cannabis/SCRAs

Adapted from the Drinking Motives Questionnaire (Cooper et al. 1992), this tool provides 17 reasons for using cannabis or SCRAs (responses: 1 = never, 2 = rarely, 3 = sometimes, 4 = often, 5 = almost always) allowing selection of all applicable motivations and the most important overall. Responses are categorised into four motivational domains: social (e.g. to be sociable, to make social gatherings more enjoyable), coping (e.g. to forget worries, to increase confidence), pleasure enhancement (e.g. because it is fun, to get high) and illness (e.g. to reduce symptoms of mental illness). Higher scores indicate higher motivation.

### Mental health

Psychological distress was measured using the 21-item Depression, Anxiety, Stress Scale (DASS21:Lovibond and Lovibond 1995). Three sub-scales assess associated symptoms during the previous week. Higher scores indicate higher distress.

### Health status

General health status was assessed using the 36-item Short Form Health Survey version 2 (SF-36v2) (Ware et al. 2002). Health over a 4-week period is assessed across eight physical and mental health domains, providing two standardised global measures, the physical component and mental component summary. Higher scores suggest better quality of life.

Participants in the cannabis-only group completed:

### SCRA: no use

Designed for the study, this questionnaire specifies 14 reasons for not trying SCRAs (e.g. not natural, no curiosity, heard bad/negative reports). Multiple responses are allowed, with the most important reason specified.

In addition to those previously indicated, participants in the SCRA + cannabis group completed:

### Comparing SCRAs and natural cannabis

Participants indicated (a) which of the two drug-types was preferred overall and why and (b) which most met each of eight following criteria and why: consistency, value for money, self-rated addictive potential, positive effects when high, negative effects when high, harm to lungs, safety and long-lasting effects. Items were adapted from Winstock and Barratt (2013b).

### Reasons for first and subsequent SCRA use

Thirteen identical reasons were offered for (a) initially trying and (b) continuing with SCRA use. Multiple responses were allowed and the most important reason for both identified. All items were adapted from Barratt et al. (2013).

### Utilisation of health services

Participants are asked to specify any health-related treatment they received in relation to their SCRA use.

#### Analysis

Responses for all variables were analysed descriptively using bivariate associations and relative frequencies (with results stated as both a percentage and proportion of valid responses), means (M) with standard deviation (SD) and where appropriate, medians (Mdn) with interquartile range (IQR). Student’s *t*-test, Pearson’s chi-square, Kruskal–Wallis and Mann–Whitney *U* (U) tests were used to determine inter- and between-group differences with the magnitude of difference measured using Cohen’s *d*. McNemar tests were used for within-subject proportional comparisons, with significant results provided using odds ratio (OR) and confidence intervals (CI). Logistic regression was used to determine participant characteristics that were associated with SCRA use while controlling for other variables. Predictor variables were those between group differences that were significant in bivariate analysis (at *p* < 0.05).

Responses to the effects of SCRAs and cannabis items were categorised into ‘effect absent’ (never responses) and ‘effect present’ (sometimes, most of the time and always responses) to create paired 2 × 2 contingency tables. Conditional logistic regression established ORs with CIs for each and McNemar tests verified significant differences.

## Results

One hundred and fifty-four participants provided consent to participate and completed the interview process. Eighty-two were in the SCRA + cannabis group and 72 in the cannabis-only group. Frequencies differ in instances where participants did not provide responses.

### Demographic information

Table 1 provides demographic information. A higher proportion of cannabis-only participants reported being single (80% v 65%, *p* = 0.03) and living alone (40% v 23%, *p* = 0.02).

**Table 1:** Sociodemographic characteristics of 154 participants undergoing treatment for cannabis use in New South Wales, Australia

Demographic	SCRA + cannabis *(n* = 82) *n* (%)	Cannabis-only (*n* = 72) *n* (%)	Total (*N* = 154) *n* (%)	*p*-value
Age in years *mdn(IQR)*	32 (11)	37 (10)	35 (10)	0.17
Age range *n* (%)				
18–35	46 (56%)	34 (47%)	80 (52%)	
36 +	36 (44%)	38 (53%)	74 (48%)	0.27
Male, *n* (%)	62 (76%)	48 (67%)	110 (71%)	0.22
Aboriginal, *n* (%)	8 (10%)	8 (11%)	16 (11%)	0.76
Education ≤ year 10, *n* (%)	45 (55%)	39 (54%)	84 (55%)	0.93
Single relationship status, *n* (%)	53 (65%)	57 (80%)	110 (72%)	0.03
Live alone, *n* (%)	19 (23%)	29 (40%)	48 (31%)	0.02
Government support, *n* (%)	61 (74%)	52 (72%)	113 (73%)	0.76

### Substance use information

Eighty-four percent (130/154) described use of cannabis in the previous month, consuming an average of 2.2 g/day on 19/28 days. Eighty-eight percent (135/154) met ICD-10 criteria for cannabis dependence. Average age of cannabis initiation was 14.4 (SD = 3.0). Nearly all (89%) used water pipes as their main route of administration but use of ‘joints’ (92%; 142/154), dry pipe (82%; 126/154), ingestion (77%; 118/154) and vaping (29%; 44/154) were also described.

Table 2 describes differences between groups in cannabis use characteristics. The cannabis-only group consumed significantly more cannabis (2.8 g vs 1.7 g, *p* = 0.03) more regularly (21/28 vs 16/28, *p* = 0.01) and reported more symptoms of cannabis dependence (Mdn(IQR) = 6(1) vs 5(2), *p* ≤ 0.01) than those from the SCRA + cannabis group. The SCRA + cannabis group were more likely to have ingested cannabis orally (71/82 vs 46/72, *p* ≤ 0.01) or smoked via a dry pipe (73/82 vs 53/72, *p* = 0.02) than the cannabis-only group.

**Table 2:** Differences in cannabis use characteristics between the SCRA + cannabis group and the cannabis-only group participants

Cannabis use characteristic	SCRA + cannabis *(n* = 82)	Cannabis-only (*n* = 72)	*p*-value
Used in past 3 months, *n* (%)	74 (90%)	68 (94%)	0.33
Used in past month, *n* (%)	63 (88%)	67 (91%)	0.32
Number of days used in past month, *m(sd)*	16.4 (11.2)	21.0 (10.3)	0.01
Average amount used per day (grammes), *m(sd)*	1.7 (1.8)	2.9 (4.5)	0.03
Age of initiation (years), *m(sd)*	14.2 (3.0)	14.7 (2.9)	0.33
No. of ICD-10 symptoms, *mdn(IQR)*	5 (2)	6 (1)	< 0.01
Route of administration:			
Water pipe, *n* (%)	71 (99%)	82 (100%)	0.28
Joints, *n* (%)	77 (94%)	65 (90%)	0.14
Dry pipe, *n* (%)	73 (89%)	53 (74%)	0.02
Vaporiser, *n* (%)	27 (33%)	17 (24%)	0.27
Ingested, *n* (%)	72 (88%)	46 (64%)	< 0.01

Approximately half reported previous use of SCRAs (53%; 82/154), with 20% reporting previous month consumption (16/82) with the median being 20 days (IQR = 27). Half (50%; 8/16) reported daily or almost daily use. Although half the sample had used SCRAs ≥ 20 times (52%; 43/82), one-third had attempted them less frequently (< 5 times–37%; 30/82). Many brands of SCRA were consumed with Kronic, Black Widow, K2 and Northern Lights the most commonly identified. The median age of first consumption was 29 years (IQR = 19). Waterpipe was the main method of ingestion (95%; 78/82) although smoking by joint (27%; 22/82) or dry pipe (27%; 22/82), as well as vaping (2%; 2/82) and injecting (2%; 2/82), was detailed. Mixing SCRAs with tobacco was also common (77%; 63/82).

Self-reported substance use and between-group comparisons for a range of substances including SCRAs are described in Table 3. Total group median lifetime use of 7.5 substances (including cannabis) (IQR = 4) and past-month use of 3 (IQR = 2) were recorded. The SCRA + cannabis group consumed a higher number of substances over a lifetime than the cannabis-only group [Mdn(IQR) = 9(4) v 6(3), U = 1427 *p* < 0.001] and were more likely to report lifetime use of hallucinogens (69.5% v 36.1%, *p* < 0.001), synthetic stimulants (32.9% v 4.2%, *p* < 0.001), inhalants (26.8% v 5.6%, *p* < 0.001), illicit opioids (39.0% v 16.7%, *p* = 0.002) and illicit benzodiazepines (25.6% v 12.5%, *p* < 0.04). The cannabis-only group were more likely to report lifetime use of prescribed opiates (47.2% v 26.8%, *p* = 0.009).

**Table 3:** Substance use history of 154 participants undergoing treatment for cannabis use in New South Wales, Australia

**Substance use**	**Total (*N* = 154)**				**SCRAs (*n* = 82)**				**Cannabis (*n* = 72)**				**Comparison of lifetime substance use**^a^ **(*p*-value)**
	**Ever** **(%)**	**3 month** **(%)**	**1 month** **(%)**	**Median** **Days/28** ***n* (IQR)**	**Ever** **(%)**	**3 month** **(%)**	**1 month** **(%)**	**Median** **Days/28** ***n* (IQR)**	**Ever** **(%)**	**3 month** **(%)**	**1 month** **(%)**	**Median** **Days/28** ***n* (IQR)**	
Cannabis	100.0	92.2	84.4	26 (18–28)	100.0	90.2	81.7	21 (4.5–26)	100.0	94.4	87.5	28 (14–28)	-
SCRA	53.2	14.3	10.4	20 (1–28)	100.0	26.8	19.5	20 (1–28)	0	0	0	0	-
Alcohol	96.1	72.1	63.6	5 (2–14)	97.6	76.8	68.3	5 (2–13)	94.4	66.7	58.3	5 (3–14)	0.319
Tobacco	89.6	80.5	78.6	28 (26–28)	90.2	82.9	79.3	28 (25–28)	88.9	77.8	77.8	28 (28–28)	0.783
Amphetamines	79.2	38.3	27.3	4 (1–20)	80.5	35.4	26.8	5 (1–20)	77.8	41.7	27.8	4 (1–11)	0.679
Ecstasy	68.2	14.9	7.1	2 (1–2)	72.0	18.3	7.3	1 (1–2)	63.9	11.1	6.9	2 (1–2)	0.284
Hallucinogens	53.9	3.9	1.9	3 (2–4)	69.5	4.9	2.4	3 (2–3)	36.1	2.8	1.4	3 (3–3)	< 0.001
Cocaine	51.9	11.0	5.8	2 (2–4)	50.0	7.3	2.4	3 (3–6)	54.2	15.3	9.7	2 (2–5)	0.606
Benzodiazepines—prescribed	39.6	24.7	22.1	3 (2–7)	45.1	29.3	26.8	3 (2–6)	33.3	19.4	16.7	5 (2–7)	0.136
Opioids—prescribed	36.4	23.4	18.8	7 (4–28)	26.8	13.4	9.8	5 (2–15)	47.2	34.7	29.2	10 (5–28)	0.009
Opioids—illicit	28.6	8.4	7.1	2 (1–8)	39.0	12.2	11.0	2 (1–7)	16.7	4.2	2.8	17 (13–21)	0.002
Synthetic stimulants	19.5	2.6	0	0	32.9	3.7	0	0	4.2	1.4	0	0	< 0.001
Benzodiazepines—illicit	19.5	7.8	7.1	2 (1–4)	25.6	12.2	11.0	2 (1–4)	12.5	2.8	2.8	3 (3–3)	0.040
Inhalants	16.9	0.6	0	0	26.8	0	0	0	5.6	1.4	0	0	< 0.001
Opioid agonist treatment	7.1	2.7	2.7	28 (28–28)	9.8	3.7	3.7	28 (28–28)	4.2	1.4	1.4	28 (28–28)	0.187
Other	6.5	0.6	0.6	1 (1–1)	9.8	1.2	1.2	1 (1–1)	2.8	0	0	0	0.080

^a^Chi-square test comparisons of SCRA and cannabis groups for ever use of all substances, excluding cannabis and SCRAs

#### Drug Use Motive Questionnaire (DUMQ): cannabis/SCRA

The primary psychological motivations to consume cannabis and SCRAs were for pleasure enhancement (M = 13 and 11.3 respectively) and as a means of coping (M = 12.9 and 7.8 respectively). Both the highest scoring item of each sub-scale and the most important reasons selected to use cannabis were ‘to relax’ (22%; 33/152) and ‘to get high’ (15%; 23/152). The most frequently chosen and most important reason for SCRAs use was ‘to get high’ (46%; 37/81), followed by ‘because friends are doing it’ (15%; 12/81).

**Fig. 1:**
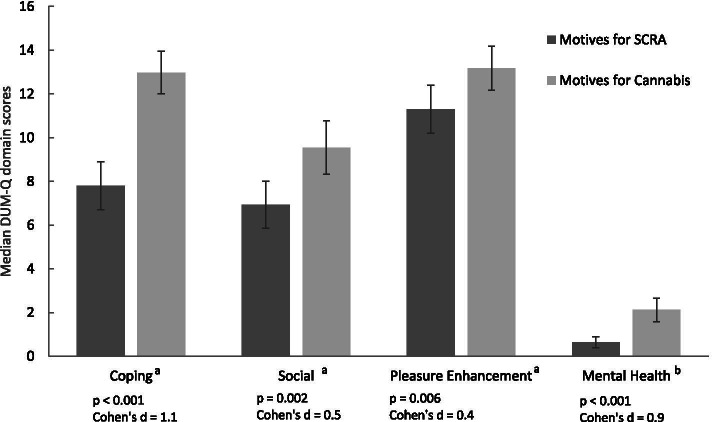
Difference in median Drug Use Motives Questionnaire (DUM-Q) domain scores for SCRAs and cannabis. **a** The Coping, Social and Pleasure Enhancement domains consist of 4 items, each with a 5-point response option scored 0 to 4 respectively (never=0, rarely=1, sometimes=2, often=3, almost always=4). **b** The Mental Health domain consists of only 2 items, each with the same response and scoring as above

There were no differences between groups in the motives for use of cannabis. Figure 1 illustrates significant differences between the SCRA + cannabis group motives for use of cannabis and SCRAs.

#### Effects of cannabis/SCRA use

Table 4 describes comparison data for self-reported side-effects experienced during or after their use of cannabis and SCRAs. Differences were reported across the spectrum including psychotic reactions and neurological and cardiovascular effects. No differences were found in the number of effects experienced between genders [M (SD) = 12.5(7) male vs 10.9(6.5) female, *p* = 0.36].

**Table 4:** Comparison of effects of SCRA and cannabis use as reported by 154 participants undergoing treatment for cannabis use in New South Wales, Australia

Self-reported effects/experiences^a^	SCRAs (%) *(n* = 82)	Cannabis (%) *(n* = 72)	OR	95% CI	McNemar Test *p*-value
Racing heart/irregular heartbeat	40.2	9.8	4.1	1.9–8.9	< 0.001
Nausea	37.8	9.8	3.9	1.8–8.4	< 0.001
Panic	37.8	17.1	2.2	1.1–4.2	0.016
Dizziness	35.4	9.8	3.6	1.7–8.0	0.001
Excessive Sweatiness	31.7	9.8	3.3	1.5–7.2	0.003
Hangover effect	31.7	12.2	2.6	1.3–5.4	0.011
Dream-like state	31.7	22.0	1.4	0.8–2.6	0.291
Chest pain	28.0	8.5	3.3	1.4–7.7	0.005
Hallucinations	28.0	7.3	3.8	1.6–9.4	0.002
Decreased appetite	25.6	15.9	1.6	0.8–3.2	0.229
Light headedness	24.4	11.0	2.2	1.0–4.9	0.061
Confusion/disorientation	24.4	9.8	2.5	1.1–5.7	0.036
Psychotic experiences	20.7	8.5	2.4	1.0–5.9	0.064
Headache	20.7	11.0	1.9	0.8–4.2	0.169
Paranoia	19.5	13.4	1.5	0.7–3.1	0.442
Decreased motor coordination	19.5	12.2	1.6	0.7–3.5	0.327
Decreased ability to function after use	18.3	15.9	1.2	0.6–2.4	0.851
Nervousness or anxiety	18.3	12.2	1.5	0.7–3.4	0.424
Feelings of personal isolation	18.3	14.6	1.3	0.6–2.7	0.701
Nausea or vomiting	18.3	19.5	0.9	0.5–1.9	1.000
Passed out after use of SCRAs	17.1	14.6	1.2	0.5–2.5	0.845
Felt antisocial after smoking	17.1	13.4	1.3	0.6–2.8	0.690
Slurred Speech	17.1	11.0	1.6	0.7–3.6	0.405
Ringing in the ears	17.1	12.2	1.4	0.6–3.2	0.541
Worried about meeting strangers	15.9	11.0	1.4	0.6–3.4	0.523
Depression	13.6	19.8	0.7	0.3–1.5	0.442
Convulsions	12.2	4.9	2.5	0.8–8.0	0.180
Feel more energetic	11.0	34.1	0.3	0.2–0.7	0.003
Increased ability to function after use	8.5	43.9	0.2	0.1–0.4	< 0.001
Felt sluggish/heavy	7.3	18.3	0.4	0.2–1.0	0.078
Clumsy	6.1	24.4	0.3	0.1–0.7	0.004
Drowsiness	4.9	29.3	0.2	0.1–0.5	< 0.001
Dry mouth	2.4	23.2	1.1	0.0–0.5	< 0.001
Impaired memory	2.4	23.2	0.1	0.0–0.5	< 0.001
Felt more focused than usual	1.2	57.3	0.0	0.0–0.2	< 0.001

^a^Every item has four response options (1 = never, 2 = sometimes, 3 = most of the time, 4 = every time), with each answer categorised into ‘effect absent’ for never responses and ‘effect present’ for sometimes, most of the time and always

#### Mental health

The DASS21 scored the overall group’s psychological health in the moderate range for depression and anxiety and the mild range for stress [Mdn (IQR) = 8(8.5), 5(7), 8(9) respectively]. The SCRA + cannabis group indicated higher levels of stress [Mdn (IQR) = 10(9.5) v 6.5(9), U = 2270, *p* = 0.01] and psychological distress overall [Mdn (IQR) = 8(9) v 6(9), U = 23,064 *p* = 0.01] compared to the cannabis-only group. See Supplementary Materials for DASS scoring.

#### Health status

Between-group comparisons were similar in all subscales except role physical, with the cannabis-only group indicating greater role limitations due to physical health concerns (M (SD) = 41.4(8.7) v 50(10.2), *p* < 0.001). Likewise, their global physical component score indicated poorer overall physical health (M (SD) = 50.2(6.3) v 54.8(7.9), *p* < 0.001). Full sample data comparisons with Australian age-matched SF-36 normative data showed significantly lower functioning for both groups across all subscales except physical functioning. The global component physical score indicated no differences (M (SD) = 52.7(7.6) v 52.7(7.8), *p* = 0.41), but the mental component score was significantly lower when compared to population norms (M (SD) = 34.7(12.2) v 49.4(9.6), *p* < 0.001).

### SCRA use

#### Predictors of SCRA use

Results of bivariate logistic regression analysis for individual demographic, substance use and psychological wellbeing characteristics significantly associated with SCRA use are demonstrated in Table 5. These indicate that living alone, being single and having more symptoms of cannabis dependence decreased the odds of having used SCRAs, while consuming a greater number of substances increased the odds of SCRA use by 40%.

**Table 5:** Logistic regression model predicting SCRA use in an Australian cannabis treatment population (*N* = 154)

Characteristics	SCRA use		
	**OR**	**95%CI**	***p-value***
Living arrangements—living with others	0.3	0.1–0.8	0.011
Relationship status—in a relationship	0.4	0.2–0.9	0.020
Cannabis dependence—ICD-10 score	0.8	0.6–0.9	0.012
Cannabis consumer/day—grammes	0.9	0.8–1.0	0.083
Lifetime substance use (excluding cannabis and SCRAs)	1.4	1.1–1.6	0.001
DASS—stress score	1.1	1.0–1.3	0.180
DASS—overall score	1.0	0.9–1.0	0.761

#### First, subsequent and no use

Table 6 illustrates reasons for first and subsequent SCRA use and selection of the most influential reasons for both. It also details the cannabis-only group’s reasons not to use SCRAs.

**Table 6:** Reasons for first, subsequent use and no use of SCRAs with most important reason for each highlighted and italicised

First SCRA use (*n* = 82)	Reasons for SCRA use n, %		Most important reason n, %	
***Curiosity to compare effects to cannabis***	63	77%	***18***	***22%***
Offered by others/available	51	62%	15	18%
Heard reports from other sources	50	61%	7	9%
Offered an alternative to cannabis	42	51%	13	16%
Legal status	42	51%	10	12%
Easier to obtain than cannabis	31	38%	4	5%
Subject to drug testing	20	24%	7	9%
To reduce or cease cannabis use	15	18%	6	7%
Produced desirable recreational effects	14	17%	-	-
Less expensive than cannabis	10	12%	1	1%
Stronger than cannabis	10	12%	1	1%
Produced positive therapeutic/medicinal effects	7	9%	-	-
Milder than cannabis	4	5%	-	-
**Subsequent SCRA use (*n* = 72)**				
Offered an alternative to cannabis	37	51%	7	10%
Easier to obtain than cannabis	34	47%	7	10%
***Stronger than cannabis***	33	44%	***13***	***18%***
Legal status	31	43%	7	10%
Offered by others/available	31	43%	9	13%
Produced desirable recreational effects	22	31%	6	8%
Subject to drug testing	20	28%	9	13%
Curiosity to compare effects to cannabis	19	26%	9	13%
Produced positive therapeutic/medicinal effects	10	14%	-	-
Less expensive than cannabis	9	13%	3	4%
To reduce or cease cannabis use	8	11%	2	3%
Heard reports from other sources	8	11%	-	-
Milder than cannabis	5	7%	-	-
**No SCRA use (*n* = 72)**				
***Heard bad/negative reports***	62	86%	***37***	***51%***
Not a natural product	50	69%	17	24%
No curiosity to try SCRAs	40	56%	5	7%
Undesired recreational effects	38	53%	7	10%
Distrust of SCRA manufacturers	20	28%	2	3%
Never offered or available	14	19%	4	6%
Negative/harmful effect profile	14	19%	-	-
Distrust of SCRA retailers	11	15%	-	-
Illegal status	9	13%	-	-
More difficult to obtain	8	11%	-	-
Stronger than cannabis	5	7%	-	-
Subject to drug testing	3	4%	-	-
More expensive	2	3%	-	-
Milder than cannabis	2	3%	-	-

#### Comparing SCRAs and cannabis

Of the SCRA + cannabis group, 89% (73/82) preferred cannabis. Most felt that it provided a safer experience (98%; 80/82), had more positive psychoactive effects (91%; 75/82), had longer lasting effects (79%; 65/82), offered a more consistent product (72%; 59/82) and was better value for money (66%; 54/82). In terms of side-effects, most participants considered SCRAs to be more harmful to the lungs than cannabis (78%; 64/82) and to have more negative psychoactive effects (91%; 75/82). When asked which of the two they would offer a novice substance consumer, SCRA participants overwhelming elected cannabis (93%; 76/82). For those who preferred SCRAs, rationales included their superior psychoactive strength and quality, their ability to evade drug testing and ease of accessibility.

#### Utilisation of health services

Of the SCRA + cannabis group, 27% (22/82) sought medical assistance in relation to SCRA use. Of these, 45% (10/22) were admitted for hospital treatment and 32% (7/22) were transported by ambulance. Reasons for hospital admission included: detoxification (50%; 5/10), psychosis (30%; 3/10), overdose (10%; 1/10) and seizures (10%; 1/10). Treatment by other medical professionals included psychologist/ counsellor (77%; 17/22), medical practitioner (59%; 13/22), psychiatrist (27%; 6/22) and neurologist (14%; 3/22).

## Discussion

The current study examines SCRA use among treatment groups of cannabis consumers in metropolitan and regional areas of New South Wales, Australia. The cohort (*n* = 154) predominantly consisted of older [Mdn (IQR) = 35(18)], single, socioeconomically disadvantaged, cannabis-dependant males. As expected, use of multiple substances and high levels of psychological distress were recorded, with overall mental health significantly lower than that of the Australian population in the same age range.

The SCRA + cannabis group were largely male, although somewhat older [Mdn (IQR) = 32(17)] than those described in earlier research (Winstock and Barratt 2013b; Barratt et al. 2013). This is consistent with recent Australian data (Grigg et al. 2020) and that from a nationwide retrospective case-series of deaths related to SCRA consumption between 2000 and 2017 (Darke et al. 2020). Here, those suffering fatal harm (*n* = 55) were male (91%) and older age [M (SD) = 37(12)] with over two-thirds having a documented history of cannabis use. Older age was associated with death due to combined toxicity and cardiovascular disease, suggesting groups such as those presenting for cannabis-related treatment may be at greatest risk of acute harm. US data on SCRA use in substance treatment populations is also consistent, with SCRA consumers being similar age (M = 32.5) although younger than those who had not used SCRAs (M = 40.7). They too were predominantly single (60.3%) with extensive substance use histories (Smith and Staton 2019).

### SCRA prevalence and use

SCRA use was relatively common, with approximately half the total sample reporting lifetime use. Despite decreases in SCRA availability and use in Australia (Downey 2014), almost 20% described previous month use and 10% reported daily use.

Our research shows that an individual’s relationship with cannabis and other substances may influence SCRA use. Those less dependent on cannabis with experience using a wide range of other substances are more likely to be SCRA consumers. The SCRA + cannabis group were more socially integrated and exhibited greater poly-substance use, but were more stressed and had poorer mental health than their cannabis-only counterparts. This demonstrated use of SCRA by disadvantaged groups supports growing literature that psychosocially vulnerable communities, characterised by poly-substance use, are more likely to consume SCRAs and suffer associated harms (Manseau et al. 2017; Joseph et al. 2019; Sutherland et al. 2018). The results also align with suggestions that SCRA use is most likely in regular cannabis and amphetamine consumers (Sutherland et al. 2018).

### Motivations to use SCRAs

Pleasure enhancement and coping were the most common reasons to consume SCRAs and cannabis. Curiosity was the motivation for initial SCRA use, as described in earlier studies (Bonar et al. 2014; Vandrey et al. 2012; Barratt et al. 2013). The circumvention of drug testing has previously been noted as a significant motive to use SCRAs (Bonar et al. 2014; Gunderson et al. 2014; Vandrey et al. 2012), but only one-quarter of the current SCRA + cannabis group cited this. High levels of unemployment, reducing exposure to workplace drug testing, may partly explain this difference. Reasons to continue SCRA use centred on the strength of their psychoactive effects: this response has not been highlighted in SCRA literature but does support a descriptor of NPS consumers as an innovative group of people who use substances and actively seek altered states of consciousness (Sutherland et al. 2016).

### Side effects

A range of side-effects of SCRA intoxication are described, many overlapping with those seen after high-dose cannabis intoxication (Fattore 2016). The present results suggest that SCRAs are more likely to induce negative side-effects (rapid heartbeat, nausea, hallucinations, chest pain, sweatiness, dizziness, and panic) than cannabis, although survey design limitations should be acknowledged. The rating scale fails to account for the relativity frequency of events and how they correspond with given response options. For example, an effect experienced three times may be rated as occurring ‘sometimes’ after 100 uses of cannabis, but ‘nearly always’ after five uses of SCRA. Consistent with other studies, only few participants reported experiencing regular post-SCRA consumption psychotic symptoms and hallucinations (Barratt et al. 2013; Vandrey et al. 2012). Some effects may be difficult to attribute due to variability in the psychoactive ingredients used in SCRA products. Age, gender and concurrent use of other substances may also impact the number and severity of self-reported effects (Fattore 2016; Barratt et al. 2013).

### Medical treatment

One-quarter of the respondent’s required medical treatment to address short- or long-term consequences of SCRA use. Of these, one in 20 sought emergency hospital treatment, a rate exceeding the one in 40 SCRA-related emergency medical presentations estimated by Winstock et al. (2015) (Winstock and Barratt 2013a). It is possible that the current sample, who were already experiencing problems with substance use, would be more likely to seek treatment than those who use substances and respond to internet surveys. Several other factors could also be accountable, such as age differences between survey and study respondents [Mdn (IQR) = 23(9) vs 32(17) respectively] or time differences between data collections (2011 vs 2015 respectively), and changes in SCRA diversity in that time. The latter is consistent with recent Australian data indicating increasing probability of requiring presentation to emergency departments following SCRA consumption between 2015 and 2018 (Grigg et al. 2020) implying a pattern of escalating SCRA toxicity or more acute clinical presentation. The pharmacological literature indicates increasing SCRA potency over time, with higher numbers of recent compounds having sub nanomolar affinity at CB1 receptors (Banister S.D. and Connor M. 2018).

All hospital presentations related to serious SCRA side effects or withdrawal management rather than less serious adverse events (e.g. panic/anxiety, tachycardia or agitation) (Tait et al. 2016). Previous treatment experiences for cannabis-related problems were not explored, although exposure to SCRAs has already been shown to significantly increase the risk of short-term harms when compared to cannabis (Winstock et al. 2015; Winstock and Barratt 2013a). Comparisons with previous findings are limited by the lack of timeframes and variety of treatment services included in the current study. Nevertheless, a concerning incidence of serious harm is highlighted.

### Substance preferences

In agreement with previous findings (Winstock and Barratt 2013b), an overwhelming majority of respondents preferred cannabis to SCRAs. Explanations centred on psychoactive effects (both dissatisfaction with those of SCRAs and preference for the effects of cannabis), the ‘natural’ verses ‘synthetic’ nature of the two substances and safety concerns about the use of SCRAs. The preference perhaps explains some of the decline in SCRA use seen in general populations in Australia in recent years. It is notable that despite this, SCRA use was neither uncommon nor infrequent in this group.

### Strengths and limitations

This is the first Australian exploration of SCRA use in a treatment sample of people who use cannabis. Its strength lies in the face-to-face method of information collection that allowed exploration of a population that can be difficult to capture using online survey techniques. The results are broadly consistent with previous findings and provide new evidence regarding the impacts of SCRA use in substance use treatment populations.

The interview data was subject to recall bias. No toxicology screening or verification measures were undertaken, so outcomes attributed to SCRAs may be due to unrelated factors. It should also be noted that SCRAs are a broad class of substance with a wide-ranging array of SCRA product types, so consumer experiences cannot be attributed to a homogenous drug type.

The results have limited external validity given the groups specific nature and high levels of comorbid mental illness and substance use. Lastly, the relatively small sample size may limit opportunities of detecting more subtle group differences, although a range of robust findings emerged nonetheless.

## Conclusions

SCRAs are one sub-category within the broad range of NPS with potential for toxicity and long-term harms to users. The study provides a picture of SCRA use in Australian cannabis treatment populations. More than half of those receiving treatment for cannabis use report lifetime SCRA use and despite changing availability, approximately one-quarter described recent SCRA use. Having a lower dependence on cannabis but higher level of other substance use may predict SCRA use. Motivations for initial SCRA consumption were curiosity but psychoactive strength precipitated continued use even though cannabis was overwhelmingly preferred by this group. Overall, SCRAs appear to induce more negative side-effects than cannabis and seeking medical assistance, including hospital admissions, was relatively common.

Those who use SCRAs are likely to experience greater poly-substance use and psychological distress than those who do not and as such these substances remain a health concern for vulnerable groups of socially disadvantaged individuals with substance use and/or mental health concerns. Future research should monitor the prevalence of even low-level SCRA and NPS use among these individuals to identify changes in availability and consumption. Clinically, the need to monitor SCRA consumption in individuals persists as identification of its use alongside other illicit drug use may indicate more severe or problematic patterns of substance use and/or mental health concerns requiring treatment.

## Supplementary Information

**Additional file 1**. Supplementary Material.

## Data Availability

The data that informs the findings of this study is available from the corresponding author (MAJ) upon reasonable request.

## Abbreviations

NPS: New psychoactive substances
SCRA: Synthetic cannabinoid receptor agonists
NSW: New South Wales
M: Median
SD: Standard deviation
Mdn: Median
IQR: Interquartile range
*U*: Mann–Whitney *U*
OR: Odds ratio
CI: Confidence interval
