# Supplementary material for: The use and effects of synthetic cannabinoid receptor agonists by New South Wales cannabis treatment clients
Source: J Cannabis Res. 2021 Jul 26;3:33. doi: 10.1186/s42238-021-00091-z (PMC8314558; doi:10.1186/s42238-021-00091-z)
Supplement: Supplementary file 1 — Additional file 1. Supplementary Material. [file 42238_2021_91_MOESM1_ESM.docx]

## Supplementary Material

### Measures - additional detail

#### Substance Use History

This questionnaire provides a retrospective review of client substance use and was adapted from that utilised by clinical treatment services of New South Wales Health. Respondents report lifetime, 3-month and 1-month use, including the number of days used in the month, of a range of illicit and prescribed substances, including synthetic cannabinoids.

#### Cannabis dependence

Cannabis dependence is assessed using the ICD-10 [1]. The 7-item questionnaire identifies a dependence syndrome based on: (a) compulsion to use cannabis; (b) inability to control consumption; (c) withdrawal symptoms & their avoidance; (d) tolerance; (e) continued use despite social interference; and (e) continued use despite significant harms. Dependence is considered if three or more of these six criteria had been experienced in the previous 12-month period.

#### Effects of cannabis/SCRA use

Thirty-five physiological, psychological and behavioural consequences of cannabis and synthetic cannabinoid use were rated by the extent to which respondents had experienced each of them while intoxicated (1=Never, 2=Sometimes, 3=Most of the time, 4=Every time). Designed specifically for the current study, the survey incorporated a range of effects (e.g. felt stimulated/energetic; increased ability to function; felt nervous or anxious; had a dry mouth). Items were selected based on previous literature [2-4] and assessed for face-validity* by study investigators AD, NL, DA, JJ, RC and RB.

#### Drug Use Motive Questionnaire (DUMQ)

Adapted from the Drinking Motives Questionnaire [5], this questionnaire allows assessment of motives for substances other than alcohol [6]. Seventeen potential reasons for using cannabis or synthetic cannabinoids are offered with five response options (1=Never, 2=Rarely, 3=Sometimes, 4=Often, 5=Almost Always) and the most important reason was selected. Responses were categorised into four motivational domains: social (e.g. to be sociable); coping (e.g. to forget worries); pleasure enhancement (e.g. because it is fun); and illness (e.g. to reduce symptoms associated with mental illness). Illness was added to the original 15-item version to increase its relevance to those with mental disorders. Confirmatory factor analysis of the 17-item DUMQ for cannabis has supported the four-factor model and its use with clinical samples. Internal consistency has shown to be satisfactory (Social α = .78 to 83; Coping, α = .77 to .84; Pleasure Enhancement, α = .83 to .86; Illness, α = .47 to .61); illness motive values are lower due to its representation by only two items [6].

#### DASS:21

Psychological distress was measured using the short-form Depression, Anxiety, Stress Scale [DASS21:7]. Twenty-one items assessed symptoms of depression, anxiety and stress observed during the previous seven days, with higher score indicating higher levels of distress. Validity for the DASS21 indicates high overall reliability (α = .93) and high depression, anxiety and stress sub-scale reliability [α = .93, .90, and .82 respectively:8]. Validation with cannabis-using populations also indicates high overall reliability [α = .91, .86, and .90 respectively:9]. Validation with cannabis-using populations also indicates high overall reliability [α = .91, .86, and .90 respectively:9].

Recommended cut-off scores for conventionally used severity labels (normal, moderate, severe) are listed below. Note that scoring is based on the long-form DASS:42 and that scoring on the DASS:21 must be multiplied by two to calculate the final score.

|  | Depression | Anxiety | Stress |
| --- | --- | --- | --- |
| Normal | 0-9 | 0-7 | 0-14 |
| Mild | 10-13 | 8-9 | 15-18 |
| Moderate | 14-20 | 10-14 | 19-25 |
| Severe | 21-27 | 15-19 | 26-33 |
| Extremely Severe | 28+ | 20+ | 34+ |

#### SF-36v2

General health status was assessed by the well-validated Australian version of the 36-item Short Form Health Survey version 2 (SF-36v2) [10]. Health over a 4-week period is assessed across eight physical and mental health domains (physical functioning, role physical, bodily pain, general health, vitality, social functioning, role emotion, and mental health). It provides two standardized global measures, the physical component and mental component summary. Norm-based scoring (with a mean of 50 and a standard deviation of 10) provides a score for each out of 100 that can be compared to composite Australian population scores for the same age cohort. Higher scores suggest better quality of life.

#### Comparing SCRAs and natural cannabis

Participants were asked to indicate: (a) which of the two drug-types was preferred overall; and (b) which most met each of eight specified criterion: consistency, value for money, self-rated addictive potential, positive and negative effects, harm to lungs, safety and longer-lasting effects. Although designed specifically for the study, items were based around similar ones used by Winstock & Barratt, (2013) [2] and assessed for face-validity* by study investigators AD, NL, DA, JJ, RC and RB.

#### Reasons for first and subsequent SCRA use

Thirteen potential reasons were offered for: (a) initially trying and (b) continuing with synthetic cannabinoid use. Reasons for both scenarios were identical and multiple responses were allowed before participants were asked to identify the most important reason for both. The items, listed below, were adapted from those used previously by Barratt et al., (2013) [2].

| Which of the following reasons were important in your decision to first use synthetic cannabinoids? | Select ALL that apply | Select MOST important reason | Which of the following reasons were important in your decision to continue using synthetic cannabinoids? | Select ALL that apply | Select MOST important reason |
| --- | --- | --- | --- | --- | --- |
| a. Curiosity, to compare effects to cannabis |  |  | a. Curiosity, to compare effects to cannabis |  |  |
| b. Synthetic cannabinoids are/were legal |  |  | b. Synthetic cannabinoids are/were legal |  |  |
| c. Synthetic cannabinoids easier to get than cannabis |  |  | c. Synthetic cannabinoids easier to get than cannabis |  |  |
| d. Produced desirable ‘recreational’ effects |  |  | d. Produced desirable ‘recreational’ effects |  |  |
| e. Alternative to cannabis |  |  | e. Alternative to cannabis |  |  |
| f. Synthetic cannabinoids were offered/available |  |  | f. Synthetic cannabinoids were offered/available |  |  |
| g. Produced positive ‘therapeutic/medicinal’ effects |  |  | g. Produced positive ‘therapeutic/medicinal’ effects |  |  |
| h. Subject to workplace/driver drug testing |  |  | h. Subject to workplace/driver drug testing |  |  |
| i. Heard reports from friends, others, media |  |  | i. Heard reports from friends, others, media |  |  |
| j. Synthetic cannabinoids less expensive than cannabis |  |  | j. Synthetic cannabinoids less expensive than cannabis |  |  |
| k. To reduce or cease cannabis use |  |  | k. To reduce or cease cannabis use |  |  |
| l. Synthetic cannabinoids milder than cannabis |  |  | l. Synthetic cannabinoids milder than cannabis |  |  |
| m. Synthetic cannabinoids stronger than cannabis |  |  | m. Synthetic cannabinoids stronger than cannabis |  |  |

#### Reasons for no SCRA use

Fourteen items were offered as reasons for not trying synthetic cannabinoids (e.g. synthetic cannabinoids not natural; no curiosity to compare effects to cannabis; heard bad/negative reports). Multiple reasons were permitted, and participants were asked to specify the most important reason for them. The items listed below were developed specifically for this study and assessed for face-validity* by study investigators AD, NL, DA, JJ, RC and RB.

| Why did you not ever use synthetic cannabinoids? | Select all that apply | Select MOST important reason |
| --- | --- | --- |
| 1. No curiosity, not interested in comparing effects to cannabis |  |  |
| 1. Synthetic cannabinoids are/were illegal |  |  |
| 1. Synthetic cannabinoids more difficult to get than cannabis |  |  |
| 1. Produced undesirable ‘recreational’ effects |  |  |
| 1. Synthetic cannabinoids were never offered/ were unavailable |  |  |
| 1. Produced negative ‘therapeutic/medicinal’ effects |  |  |
| 1. Subject to workplace/driver drug testing |  |  |
| 1. Heard bad/negative reports from friends, others, media |  |  |
| 1. Synthetic cannabinoids more expensive than cannabis |  |  |
| 1. Synthetic cannabinoids milder than cannabis |  |  |
| 1. Synthetic cannabinoids stronger than cannabis |  |  |
| 1. Synthetic cannabinoids not natural |  |  |
| 1. Don’t trust synthetic cannabinoid manufacturers |  |  |
| 1. Don’t trust synthetic cannabinoid retailers/sellers |  |  |
| 1. Other (please specify) |  |  |

*** Assessment of Face Validity:**

Face Validity was assessed by the degree to which experts judged instrument items to be appropriate for the instruments objectives. The investigators involved are considered to be experts in both the study and treatment of cannabis use and substance use more broadly. Instrument items were judged to be valid if all investigators agreed that they clearly represented the construct being measured.

## References

[1] World Health Organization. International Classification of Diseases-Tenth Revision (ICD-10). Geneva, Switzerland: Author; 1992.

[2] Winstock AR, Barratt MJ. Synthetic cannabis: a comparison of patterns of use and effect profile with natural cannabis in a large global sample. Drug Alcohol Depend. 2013;131(1-2):106-11.

[3] Copeland J, Gilmour S, Gates P, Swift W. The Cannabis Problems Questionnaire: factor structure, reliability, and validity. Drug Alcohol Depend. 2005;80(3):313-9.

[4] Vandrey R, Dunn KE, Fry JA, Girling ER. A survey study to characterize use of Spice products (synthetic cannabinoids). Drug Alcohol Depend. 2012;120(1-3):238-41.

[5] Cooper M, Russell M, Skinner JB, Windle M. Development and validation of a three-dimensional measure of drinking motives. Psychological Assessment. 1992;4(2):123-32.

[6] Thornton LK, Baker AL, Lewin TJ, Kay-Lambkin FJ, Kavanagh D, Richmond R, et al. Reasons for substance use among people with mental disorders. Addictive behaviors. 2012;37(4):427-34.

[7] Lovibond SH, Lovibond PF. Manual for the Depression Anxiety Stress Scales. 2nd ed. Sydney: Psychology Foundation of Australia; 1995.

[8] Henry JD, Crawford JR. The 21-item version of the Depression Anxiety Stress Scales (DASS–21): Normative data and psychometric evaluation in a large non-clinical sample. Br J Clin Psychol. 2005;44:227-39.

[9] Rooke SE, Norberg MM, Copeland J. Successful and unsuccessful cannabis quitters: Comparing group characteristics and quitting strategies. Substance Abuse Treatment, Prevention & Policy. 2011;6(30):1-9.

[10] Ware JE, Kosinski M, Dewey JE. How to score Version 2 of the SF-36 Health Survey (Standard and Acute forms). 3rd ed. Lincoln, RI: Quality Metric; 2002.
